# Assessing COVID-19 testing strategies in K-12 schools in underserved populations: study protocol for a cluster-randomized trial

**DOI:** 10.1186/s12889-022-13577-z

**Published:** 2022-06-13

**Authors:** Samantha Hayes, Sara Malone, Brittany Bonty, Nancy Mueller, Summer M. Reyes, Sydney A. Reyes, Christina Evans, Myisha Wilcher-Roberts, Tremayne Watterson, Sewuese Akuse, Jamee Shelley, Grace Yuan, Ian Lackey, Jasmine Prater, Brock Montgomery, Cynthia Williams, Sheretta T. Butler-Barnes, Kelly Harris, Charlene Caburnay, Nikole Lobb Dougherty, Jingxia Liu, Albert Lai, Julie Neidich, Stephanie Fritz, Jason G. Newland

**Affiliations:** 1 Department of Pediatrics, Washington University in St. Louis School of Medicine, St. Louis, MO USA; 2 Brown School, Washington University in St. Louis, St. Louis, MO USA; 3 Department of Occupational Therapy, Washington University in St. Louis School of Medicine, St. Louis, MO USA; 4 Department of Surgery, Washington University in St. Louis School of Medicine, St. Louis, MO USA; 5 Department of Medicine, Washington University in St. Louis School of Medicine, St. Louis, MO USA; 6 Department of Pathology and Immunology, Washington University in St. Louis School of Medicine, St. Louis, MO USA

**Keywords:** COVID-19, SARS-CoV-2, Testing strategies, Underserved, Community, Public health, Cluster non-randomized trial, Community-based participatory research, Under-resourced community

## Abstract

**Background:**

Since March 2020, COVID-19 has disproportionately impacted communities of color within the United States. As schools have shifted from virtual to in-person learning, continual guidance is necessary to understand appropriate interventions to prevent SARS-CoV-2 transmission. Weekly testing of students and staff for SARS-CoV-2 within K-12 school setting could provide an additional barrier to school-based transmission, especially within schools unable to implement additional mitigation strategies and/or are in areas of high transmission. This study seeks to understand the role that weekly SARS-CoV-2 testing could play in K-12 schools. In addition, through qualitative interviews and listening sessions, this research hopes to understand community concerns and barriers regarding COVID-19 testing, COVID-19 vaccine, and return to school during the COVID-19 pandemic.

**Methods/design:**

Sixteen middle and high schools from five school districts have been randomized into one of the following categories: (1) Weekly screening + symptomatic testing or (2) Symptomatic testing only. The primary outcome for this study will be the average of the secondary attack rate of school-based transmission per case. School-based transmission will also be assessed through qualitative contact interviews with positive contacts identified by the school contact tracers. Lastly, new total numbers of weekly cases and contacts within a school-based quarantine will provide guidance on transmission rates. Qualitative focus groups and interviews have been conducted to provide additional understanding to the acceptance of the intervention and barriers faced by the community regarding SARS-CoV-2 testing and vaccination.

**Discussion:**

This study will provide greater understanding of the benefit that weekly screening testing can provide in reducing SARS-CoV-2 transmission within K-12 schools. Close collaboration with community partners and school districts will be necessary for the success of this and similar studies.

**Trial Registration:**

NCT04875520. Registered May 6, 2021.

## Background

The coronavirus disease 2019 (COVID-19) pandemic has illuminated the disproportionate social and health disparities present within US communities of color. The elevated case rates, increased deaths associated severe acute respiratory syndrome coronavirus-2 (SARS-CoV-2) infection and decline in economic opportunities are just a few of the repercussions experienced within these communities [1, 2]. For children, a return to in-person learning has been met with hesitancy due to the concerns of COVID-19 transmission that in-person learning could pose.

Evidence demonstrates that school-based transmission of SARS-CoV-2 is low in the presence of COVID-19 mitigation strategies [3, 4]. Schools that have effectively implemented interventions such as social distancing, hand hygiene, masking, and increasing ventilation, have shown limited school-based transmission [3–6]. The US Centers for Disease Control & Prevention (CDC) school guidelines promote weekly screening testing as an important preventative measure within K-12 schools, especially when other prevention strategies are unable to be enacted and local transmission rates are high [7]. There is limited research evaluating the impact of weekly screening testing for SARS-CoV-2 within the K-12 school setting. There is also limited data evaluating the testing and vaccine barriers faced within predominant communities of color.

This study will assess whether weekly screening testing decreases SARS-CoV-2 transmission in middle and high schools. Additionally, the school communities’ concerns regarding in-person learning, testing, and vaccinations as it relates to the COVID-19 pandemic will be assessed.

## Methods

### Study setting and population

Five school districts within the north part of St. Louis County have been included. Students, school staff, and their household members are eligible to participate in the study. Sixteen middle and high schools from these school districts are participating. In these districts, 50–99% are comprised of minority communities and 18%-42% of families live below the poverty line [8] (Table 1). Additionally, all schools in this study receive Title 1 funding and have 100% of their students receiving free and reduced lunch. The percent of in-person attendance during the 2020–2021 school year was 20%-42%.

**Table 1:** School Demographic Characteristics

	District 1	District 2	District 3	District 4	District 5
**# of Students/Staff (# of students in-person)**					
Middle School Students	800 (160)	420 (120)	600 (240)	2590 (905)	19 (1261)
Middle School Staff	44	40	75	270	213
High School Students	650	720 (300)	800	2900 (1015)	83 (1762)
High School Staff	54	65	100	325	256
**Student Race/Ethnicity (%)**					
Black	95%	98%	83%	84%	35.1%
White	2%	1%	9%	6%	40.0%
Hispanic/Latinx	0%	0%	4%	3.5%	11.4%
Multi-racial	2%	1%	3%	4%	9.6&
**Median Household Income**	$31,646	$37,361	$59,449	$49,209	$60,732

### Study design

**Fig. 1:**
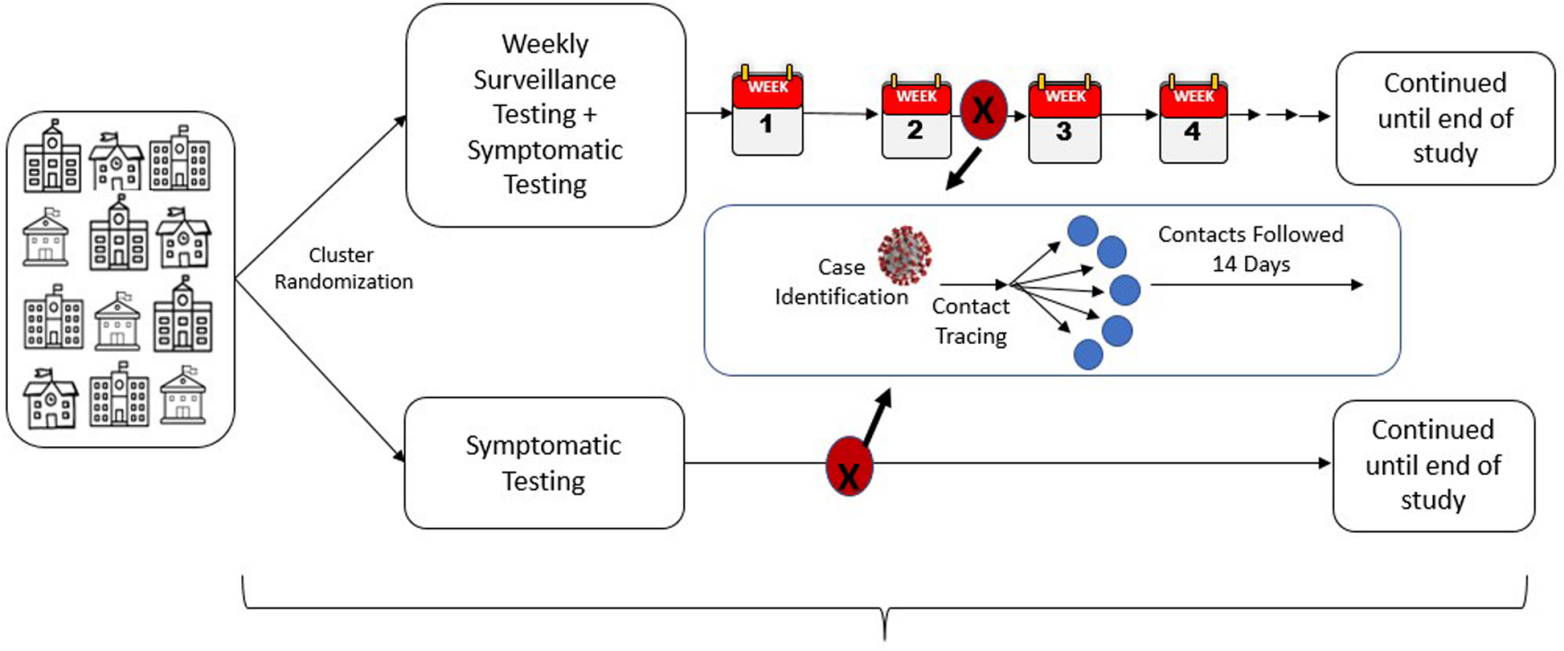
Cluster randomized trial design being utilized to assess if weekly screening testing can further limit COVID-19 transmission

A cluster randomized trial is being conducted during the 2021–2022 school year (Fig. 1). This design was chosen due to the ease of ability to directly compare interventions between schools and to mitigate allocation and selection bias within the participants. Sixteen middle and high schools within the five school districts were randomized 1:1 into one of two interventions. The first intervention was available to all schools within the 5 districts (including early learning centers and elementary schools) and consisted of access to free symptomatic or exposure-based testing. The second intervention was weekly screening based COVID-19 testing for the students, staff, and their household members plus the ability to receive symptomatic or exposure testing when needed. Symptomatic and exposure testing began in May of 2021 while the duration of the weekly screening testing intervention has been occurring during 2021–2022 school year.

A qualitative study is being conducted concurrently with COVID-19 testing (described above) to provide a deeper understanding of the community’s experiences and concerns about the pandemic, testing, and vaccinations. Focus groups are being conducted with students, parents/caregivers, and staff. School administrators are participating in semi-structured interviews.

This trial was registered to ClinicalTrials.gov on May 6, 2021. The Washington University in St. Louis School of Medicine institutional Review Board (IRB) has approved this study (202,104,013). Any important protocol changes will first be submitted to the IRB and then will be disseminated appropriately to study participants, investigators, journals, and other stakeholders.

## Community engagement

To better serve the communities of the study, we have partnered with local federally qualified health centers and community development and social service organizations. These organizations have been providing guidance regarding the best messaging and communication strategies for study participation and barriers to testing throughout the study. They have also aided us in recruitment for both testing and listening sessions and helped testing referrals and convening listening sessions.

### Community Advisory Board (CAB)

This project’s success relies on these community partnerships and our maintenance of trust with these school communities. A Community Advisory Board (CAB) has been developed to review study procedures to help ensure our study is conducted appropriately. The CAB has been fundamental in addressing the following issues: barriers to testing, participant retention, communication with the community, beliefs and attitudes about COVID-19, and other study-related activities. The CAB has met monthly and is comprised of parents, students, teachers, and administrators from each school districts and representatives from the community partners assisting with the project. The CAB is facilitated by research team members from the Brown School of Social Work and Public Health and Washington University School of Medicine testing team. This CAB will continue meeting throughout the entire study.

## COVID-19 testing procedures

### Recruitment

The community partners and CAB were consulted in developing a variety of effective and appropriate recruitment strategies within the school and community. The recruitment has been focused in two main areas. First, within the schools such as back-to-school events, townhall meetings, classrooms, and staff meetings to increase awareness about the study. Second, the study team has been conducting a mixed-methods approach to raise awareness about the study. Participating school districts disseminated study material to students, staff, and parents through electronic platforms, email, and meetings. One community partner has helped promote the testing and focus groups through canvassing efforts that consisted of door to door delivery of study promotional materials in two school districts.

### Testing

The Washington University saliva-based SARS-CoV-2 RT-PCR assay has been utilized in this study. This test, developed in conjunction with Fluidigm received emergency use authorization approval in August of 2020 [9]. Testing is performed in a CLIA/CAP certified laboratory and reported out through the Department of Pathology and Immunology at Washington University. The saliva-based assay requires that participants provide approximately 0.5 ml of saliva in a specified collection vial to conduct the analysis and is available for all age participants.

#### Screening testing

Eight of the sixteen schools have been randomized to receive weekly screening testing for students, staff, and their household members in addition to symptomatic testing. The site location and times within the randomized schools were arranged with school administration to occur on a weekly basis. The saliva-based testing method allows for staff, students, and their household members to collect the sample at home or school, then give it to a research team member on their designated weekday for specimen collection. Weekly screening testing has been available whenever in-person school is provided for schools randomized to this strategy.

Prior to starting screening testing, students, staff, and their household members were consented or assented in-person (group or individual discussion), by phone, or by zoom. Middle and high school students under the age of 18 provided assent (if cognitively able) for participation after consent was obtained from their parent/legally authorized representative (LAR.) Since household members/parents/LARs were not present in the school, they were able to receive a copy of the consent document prior to the consent discussion.

#### Symptomatic testing

All schools, including early learning centers and elementary schools, in the five districts have access to saliva SARS-CoV-2 polymerase chain reaction (PCR) testing for students, staff, and household members who exhibit symptoms of COVID-19 or have a known exposure. This testing has been provided for both virtual and in-person staff and students. Test samples have been collected at a designated drive-up testing site approximately five days a week. These drive-up testing sites rotate on a daily basis in the five school districts. In situations where transportation have been limited, home visits have been offered.

For eligible individuals who present to a nurse or study team member in two of the school districts, they have been informed about the testing option and if interested, consented and tested at site. Study Team members then collect samples within 24 h and transfer them to the laboratory.

For those who were only seeking symptomatic and/or exposure testing, verbal consent and assent (when appropriate) were obtained prior to testing, in addition to a copy of the consent information. Additionally, at the time of first consent, individuals complete the necessary Health Insurance Portability and Accountability Act (HIPAA) and email communication forms.

If a student is 18 to 21 years of age was not cognitively able to provide consent/assent, consent was obtained from the legally authorized representative. Children 8–17 years of age who were developmentally able will provide assent. Children age 5–7 would not be providing assent due to their age and development. The consenting/assenting process would be done virtually, in person or by phone.

#### Sample collection and processing

At the testing site(s), 0.5 ml of saliva are collected from each subject under the supervision of a trained research team member. Study team members then label and process the specimen through a Research Electronic Data Capture (REDCap) database. A study team member transports the specimen to the laboratory for processing. Negative test results are automatically sent to participants within 24–48 h of processing. For positive test results, a physician (JGN, SF) affiliated with the study calls the participant to inform them of their result prior to sending them their test report. The physicians can address questions of the participants and inform them of any potential therapies that might be indicated.

## Data

In addition to the saliva testing data, we have been obtaining surveys from the following: (1) school districts, (2) participants who performed saliva testing, and (3) eligible COVID-19 positive cases and their close contacts.

### School level data

To understand the current mitigation strategies present at each school, schools have been asked to complete a school-based mitigation strategy survey. This survey, developed in conjunction with the CDC and utilized in prior school-based investigations, included the following questions: method of instruction (e.g., hybrid, type of hybrid, fully in-person) and date implemented, masking recommendations and perceived compliance, distancing in classrooms, use of physical barriers (e.g., Plexiglas), location and processes for eating lunch, and mitigation strategies used in extracurricular activities (e.g., sports, choir, band) [5]. Any alterations to a school’s mitigation plan have been documented and will be taken into consideration during data analysis and interpretation. To understand transmission rates, schools were also asked about their quarantine rates and new school-based cases/contacts on a weekly basis.

### Participant level data

#### Testing

All individuals are asked one-time basic demographic and contact information that is needed to meet state requirements. The following information is collected from every participant during every sample collection: presence of symptoms, list of symptoms present in the past week and start date (if applicable), and date of sample collection. For individuals receiving symptomatic or exposure testing, the following additional information is obtained for each test collection: purpose for testing, their affiliation with the school (Student, Staff, or household member of student or staff), affiliated school district, and vaccination status. Test results are saved for each visit.

#### Case and contact interviews

If any of these individuals or other students/staff present in the school setting are positive for COVID-19, contact tracing has been performed by their school districts per local guidance. As part of their contact tracing, individuals have been informed that that they may be contacted by a member of the study team to see if they would like to provide additional information via participating in this study. Our research team has been contacting any cases and their known contacts in the school setting, as provided by the schools. After consent and assent (for those 8–17) is obtained, we asked a series of contact tracing questions about their behaviors and potential exposures. A separate case interview has also been conducted for any known contacts that have a subsequent positive COVID-19 test.

#### Common data elements survey

Anyone who has consented for screening testing, has a symptomatic testing performed for the purpose of this study and/or was identified during contact tracing, will be asked to complete the tier one common data elements (CDE) survey developed by the National Institutes of Health (NIH) [10]. Once consents have been obtained via phone, Zoom, email or in person, a link to a survey with the questions has been sent. All data collection forms are available from the research team.

## Focus Groups and Interviews

The main objectives of the focus groups and interviews is to understand the social, behavioral, and ethical factors influencing participants’ decisions and perceptions about: 1) the impact of COVID-19 on schooling; 2) facilitators and barriers to testing, attending in-person school, and vaccination; 3) successes and challenges experienced by school leadership regarding the return to in-person learning; and 4) the resources and supports provided. Eligible individuals consist of administrators, students, staff, and parents/caregivers in all five-school districts. Facilitation guides were developed for each target population and were reviewed by the CAB.

The participating school districts have shared a project description through multiple methods (e.g., email, electronic platforms, meetings) with parents/caregivers, school staff, and administrators to raise awareness about the study and participant recruitment. Recruitment also occurred at back-to-school events for families and students and during staff professional development trainings. Recruitment flyers with a (quick response) QR code have been disseminated. The QR code directs potential participants to a Qualtrics survey to sign up for a focus groups, along with demographic information. For the school administrator interviews, team members have shared project description directly with the administrators via email that includes a signup link.

Prior to the focus groups, interested participants have been emailed a consent information sheet. We have obtained verbal consent from the participants at the beginning of sessions in addition to providing a copy of the information sheet.

The majority of qualitative data collection occurs virtually via Zoom with a few student sessions being conducted in-person [11]. Each session is scheduled for one hour. All sessions are conducted by a trained facilitator and note taker on the study team. The sessions are recorded and transcribed. A directed thematic content analysis is being conducted using NVivo v12 [12, 13].

## Data Management

The participant’s information is protected throughout all aspects of data entry, coding, storage, and disseminated within secure, password-protected REDCap database and paper copies. Safeguards are in place to ensure data quality and participant’s confidentiality (E.g. restricted access, hot spots, warnings of missing responses, data quality reports.) For students and staff who complete informed consent, their positive results may be shared with the relevant school, after the individual has been called, notified of their positive result, and provides verbal re-confirmation of their permission to share that information.

Consent is obtained from participants for future use of biospecimens (saliva). The consent form allows participants to opt-out of having their saliva specimens stored and being contacted for future research studies including COVID-19 vaccination trials in children.

Our existing Consortium Data Reporting Unit (CDRU), in collaboration with our data manager, coordinate the submission of CDEs on COVID-19 testing-related outcomes to the Central Data Coordinating Center affiliated at Duke University and ensure compliance with federal, state, and local requirements for testing. We will comply with data sharing as mandated by the NIH and follow guidance provided by the CDCC for data management and support.

Important to this study is the monitoring of the social, ethical, and equity implications associated with the testing implementation in these underserved communities. All participants have been given the opportunity to express their concerns and identify barriers to participating in the study at time of enrollment. Additionally, a study email and phone number have been available for participants to provide feedback and voice any concerns about the project. Additionally, any concerns with the study will be reviewed by the team and reported to the institutional research office.

### Outcomes

The primary outcome is the secondary attack, defined as the ratio of the number of infected contacts to the total number of contacts for an infected participant. It is either NA or 0 for a participant without an infection. Such a definition is at the participant-level and its value is NA, (0–1). If we are unable to reliably obtain this information due to rule changes in quarantining or the lack of case and contact investigations, we will utilize the student and staff case rates collected by the school.

School-based transmission will be determined by two methods. First, all close contacts of an infectious case in the school, identified through contact tracing by school staff and administrators, have been approached to participate in the study (as described above). Those electing to participate have undergone a contact interview to understand their behaviors and potential other COVID-19 exposures. Furthermore, the school contact tracer has completed a survey on the relationship of the case to each contact that provides information on the location of the exposure (e.g., classroom, lunch, extracurricular activity), distance between case and contact, use of barriers, masking adherence, and total amount of time the contact was exposed to the case. The contacts will be offered testing 5–7 days after a school exposure. All contacts testing positive have undergone an independent review by five study team members using the information obtained from the case interview, contact interview, and contact tracing interview to determine whether a school-based transmission has occurred. These transmission events will be classified into the following categories with standardized definitions that were developed in conjunction with the CDC: probable, possible, unlikely, and unable to determine.

The second method to evaluate school-based transmission is to utilize collected weekly data from the schools, as participation in the above individual assessments is unlikely to reach 100%. The additional data collected will include total number of new staff and student cases, total number of new contacts, and number of school-based contacts that become new cases during their quarantine period. The secondary transmission rate will be calculated based on the number of positive school-based contacts per positive case. This method will provide the most conservative/greatest estimate of school-based transmission without knowledge of an individual’s exposure to a potential household or community case.

Lastly, if contacts are not collected are we are not able to obtain from the school, we will utilize the weekly case rates at each school. All schools have been able to provide this information.

## Statistical analysis plan

### Power calculation

A cluster randomized trial (CRT) will be conducted at 16 schools that provide symptomatic SARS-CoV-2 testing to students and staff from 2021–2022 school year. Schools are randomized 1:1 to either screening testing plus symptomatic testing, or symptomatic testing only. All participants including students and staff received the same assignment within a given school. The primary outcome is transmission rate, defined as the ratio of the number of contacts testing positive for SARS-CoV-2 to total number of contacts for each school-based case. We hypothesize that schools performing screening testing will have lower transmission rates than routine symptomatic testing. We expect that transmission rates with screening testing or routine symptomatic testing will be 2% and 8%, respectively. With the assumptions that the intracluster correlation coefficient (ICC) is 0.02 and the standard deviation of transmission rate is 0.22, 8 schools in each arm with an average of 117 participants per school will achieve 80% power to detect a difference in transmission rates between the two study arms using a two-sided t-test at a significance level of 0.05 (Table 2). PASS 15.0 was used to conduct this power analysis.

**Table 2:** Power Calculation

8 schools per group		
ICC	Standard deviation	# of participant per school
0.01	0.25	52
	0.30	96
	0.33	145
	0.35	199
0.02	0.22	55
	0.23	67
	0.24	83
	0.25	105
0.03	0.20	62
	0.21	84
0.04	0.18	58
	0.19	89
0.05	0.16	44
	0.17	71

### Analysis plan

The generalized estimating equation (GEE) model with appropriate link function (e.g., identity for primary outcome) will be used to analyze the CRT data, in which the correlation among participants within each school needs to be considered. The autoregressive of first order as a working correlation structure will be used, and participants with missing values will be excluded from the GEE analysis. The GEE model includes the group indicator and other potential factors, including race/ethnicity, insurance status, age, gender, underlying diagnoses, masking, distancing, ventilation, location of transmission. Least square means for the primary outcome per group will be estimated, and the standard errors will be calculated with the GEE sandwich method when accounting for within-school correlation. All analyses will be conducted using Statistical Analysis System (SAS) (SAS Institute, Cary, NC) at the two-sided significance level of 0.05.

This work will be disseminated using multiple strategies. This will include academic publications as well as reporting out to registries, stakeholder groups, and funding agencies. Additionally, the study team will present the information to the community stakeholders and community members engaged in the research.

## Discussions

This research seeks to evaluate how effective weekly SARS-CoV-2 testing would be as a school-based mitigation strategy in preventing school-based SARS-CoV-2 transmission. The two methods, (1) screening testing + symptomatic testing and (2) symptomatic testing alone, will be compared to understand the intervention’s possible success. We believe that screening testing will cause an additional barrier against case transmission and will result in lower case rates within the implemented schools.

Weekly testing is a prevention measure against transmission that has only recently become implemented within private and public K-12 school settings in the US. Conducting and studying the efficacy weekly testing within K-12 schools is a unique opportunity that can be tremendously beneficial to public health guidance. Following close guidance to good clinical practices, in addition to the support of the affiliated schools and local communities, is crucial to the success of this and similar community-based research projects. Our testing intervention, coupled with qualitative focus groups and interviews, is well-suited to understand the efficacy of weekly SARS-CoV-2 testing in school settings, acceptance of proposed intervention, and public reception to the SARS-CoV-2 pandemic and public health involvement.

We understand that this study has several limitations that could impede its success. First, weekly testing operations depend on the in-person learning and participant’s interests within the randomized schools. Return to remote learning due to significant increases in COVID-19 transmission could result in a sudden drop of weekly testing samples. Additional schools may be identified and selected to participate if schools remain remote, or if testing volumes are lower than expected.

## Data Availability

Not applicable.

## Abbreviations

CAB: Community Advisory Board
CDC: US Centers for Disease Control & Prevention
CDE: Common Data Elements
CDRU: Consortium Data Reporting Unit
CRT: Cluster randomized trial
COVID-19: Coronavirus disease 2019
GEE: Generalized estimating equation
HIPPA: Health Insurance Portability and Accountability Act
ICC: Intracluster correlation coefficient
LAR: Legally authorized representative
NIH: National Institutes of Health
QR: Quick response
PCR: Polymerase chain reaction
REDCap: Research Electronic Data Capture
SAS: Statistical Analysis System
SARS-CoV: Severe acute respiratory syndrome coronavirus 2
WashU: Washington University in St. Louis
