# Correction: Assessing COVID-19 testing strategies in K-12 schools in underserved populations: study protocol for a cluster-randomized trial

**DOI:** 10.1186/s12889-022-14059-y

**Published:** 2022-09-01

**Authors:** Samantha Hayes, Sara Malone, Brittany Bonty, Nancy Mueller, Summer M. Reyes, Sydney A. Reyes, Christina Evans, Myisha Wilcher-Roberts, Tremayne Watterson, Sewuese Akuse, Jamee Shelley, Grace Yuan, Ian Lackey, Jasmine Prater, Brock Montgomery, Cynthia Williams, Sheretta T. Butler-Barnes, Kelly Harris, Charlene Caburnay, Nikole Lobb Dougherty, Jingxia Liu, Albert Lai, Julie Neidich, Stephanie Fritz, Jason G. Newland

**Affiliations:** 1 Department of Pediatrics, Washington University in St. Louis School of Medi- cine, St. Louis, MO USA; 2 Brown School, Washington University in St. Louis, St. Louis, MO USA; 3 Department of Surgery, Washington University in St. Louis School of Medicine, St. Louis, MO USA; 4 Department of Medicine, Washington University in St. Louis School of Medicine, St. Louis, MO USA; 5 Department of Pathology and Immunology, Washington University in St. Louis School of Medicine, St. Louis, MO USA; 6 Department of Occupational Therapy, Washington University in St. Louis School of Medicine, St. Louis, MO USA

**Correction: BMC Public Health 22, 1177 (2022)**

**https://doi.org/10.1186/s12889-022-13577-z**

In the original publication of this article the author ‘Kelly Harris’ was accidentally omitted. The original article [1] has been updated to correct this error. The updated author contribution section is listed below.

**Authors’ contributions**

SH drafted the manuscript and contributed to the logistical framework of the study. SM contributed to the development and design of the study, data collection tools, IRB approval and continual modifications, and continued quality assurance. SM substantially participated in the drafting protocol and data management. BB contributed to the development and design of the study, data collection, and the maintenance of community partnership. BM helped in solidifying partnerships with the school districts, aided in study design, and data collection. BM participated in drafting the manuscript. NM and KH contributed to the Study design and drafting of the manuscript. SAR, SMR, JP, and JS aided in acquisition of data. In addition to data collection, CE contributed to engaging community partners and maintaining these relationships. TW contributed to data management and analysis, in addition to data collection. SA and GY helped in data collection with a heavy focus in the contract tracing data. ND contributed to the drafting of the manuscript and data analysis. EL participated in the study design and provided expertise on study outcomes and data analysis plan. El participated in drafting the manuscript. AL and IL provided expertise in Informatics and data management. SF aided in designing the trial and drafting the manuscript. JS helped design the clinical trial, identify partnerships and school districts, and drafted and revised the manuscript. All authors reviewed and approved of a final draft.
